# Sociodemographic and treatment-related correlates of fatigue in breast cancer survivors at an oncology clinic in Nigeria

**DOI:** 10.3332/ecancer.2024.1659

**Published:** 2024-01-26

**Authors:** Sharif Adeniyi Folorunso, Abbas Adesina Abdus-Salam, Atara Isiah Ntekim, Afolabi Adebayo Oladeji, Mutiu Alani Jimoh, Aminat Omolara Folorunso

**Affiliations:** 1 Department of Radiology, Obafemi Awolowo University Teaching Hospital, Ile Ife 220101, Nigeria; 2 Department of Radiation Oncology, University College Hospital/University of Ibadan, Ibadan 200005, Nigeria; 3 Department of Chemical Pathology, Obafemi Awolowo University Teaching Hospital, Ile Ife 220101, Nigeria

**Keywords:** fatigue, correlates, breast cancer, survivors

## Abstract

**Background:**

Breast cancer survivors (BCS) still experience fatigue that may impair their quality of life even after completion of treatment. There is a need to understand the sociodemographic and treatment-related factors associated with this to develop relevant and effective interventions.

**Aim:**

To assess the relationship between cancer-related fatigue and sociodemographic and treatment-related factors in BCS.

**Materials and methods:**

This is a cross-sectional study involving 80 BCS attending the radiation oncology University College Hospital Ibadan. Their sociodemographic, disease and treatment characteristics were obtained. Fatigue Symptom Inventory was used to assess fatigue. A score of at least 3 on average fatigue severity item was taken as cut-off for clinically meaningful fatigue.

**Result:**

The mean age of patients was 51.5 years. The prevalence of fatigue was 22.5%. On univariate analysis, fatigue was significantly associated with younger age (*p* = 0.022), employment (*p* = 0.006), stage of the disease(*p* = 0.014), anthracycline-based chemotherapy (*p *= 0.026), last chemotherapy less than 1 year (*p* = 0.001). Using logistic regression analysis, stage (Odds ratio (OR) 5.115, 95% CI 1.029–25.438, *p* = 0.046), employment status (OR 52.224, 95% CI 3.611–755.899, *p* = 0.004) and year of last cycle chemotherapy (OR 6.375, 95% CI 1.108–36.680, *p* = 0.038) were associated with fatigue in BCS.

**Conclusion:**

About a quarter of BCS reported fatigue. Advance stage disease, employment status and receiving last course of chemotherapy less than a year are correlates of fatigue.

## Introduction

Worldwide female breast cancer is the most common cancer accounting for 11.7% of cancer diagnoses in 2020 [1]. Breast cancer is considered the leading cause of cancer death among females in developing countries [2, 3]. The story is the same in Nigeria where breast cancer poses a major morbidity and mortality burden [1, 4, 5]. With advances in cancer treatments, such as surgery, radiotherapy, chemotherapy and other systemic therapies, coupled with increasing cancer awareness and early diagnosis, it is expected that the number of breast cancer survivors (BCS) will increase significantly in years to come [6]. Though cancer treatments improve survival, it may come with some long-term health sequelae [6, 7]. Some of these are distressing and can negatively affect the quality of life of cancer survivors [2, 8]. Some cancer survivors even stated that addressing such symptoms is as important as treating the cancer itself [7].

Fatigue is a major complaint during and after cancer treatments [9]. It could occur in up to 90% of breast cancer patients receiving chemotherapy and persist in up to 25% of patients several months after chemotherapy [10, 11]. Patients and caregivers often describe fatigue as a lack of energy, vigor or vitality. The National Comprehensive Cancer Network defined ‘cancer-related fatigue (CRF) as a distressing persistent subjective sense of physical, emotional, and/or cognitive tiredness or exhaustion related to cancer or cancer treatment that is not proportional to recent activity and that interferes with usual functioning’ [8]. Fatigue in cancer patients occurs more in the morning or afternoon as opposed to that of a healthy individual which usually occur in the evening or follow no definite pattern [11].

Gaining an understanding of the mechanisms underlying this burdensome symptom is of great interest to researchers and clinicians alike, yet relatively few studies have evaluated the aetiology of fatigue or the factors that mediate it [12]. Studies have shown that sociodemographic variables like age, marital status, gender, educational and employment status are associated with fatigue in cancer survivors [13–17]. The results of most of the studies were mixed. A systematic review reported that some studies demonstrated the relationship between fatigue and these sociodemographic variables though other studies in the same review reported no association [9]. Most of the studies were not conducted among native African population, it is therefore uncertain if these findings can be generalised to BCS in Nigeria. Some studies have also assessed the effects of previous cancer treatments especially chemotherapy on fatigue in BCS though the mechanism has not been fully understood [9, 10].

In Nigeria, a study compared the magnitude of fatigue between BCS and apparently healthy individuals [11]. Another study assessed the prevalence and determinants of chronic fatigue syndrome in apparently healthy individuals in a selected community [17]. To our knowledge, no study to date has explored the correlates of fatigue in BCS. The aim of this study is to assess frequency of fatigue in BCS and the associated sociodemographic and treatment-related variables. This is important as it will help in identifying patients with high risk and prepare them for interventions that will reduce the impact of fatigue.

## Materials and methods

This was a cross-sectional study involving BCS attending the radiation oncology clinic at the University College Hospital (UCH) Ibadan, Nigeria. Participants of this study were BCS who had completed treatment for breast cancer at least 3 months prior to recruitment with no clinical evidence of disease according to their most recent follow-up contact. Other inclusion criteria included afebrile patients (body temperature less than 37.5°C), at least 18 years of age, with no known history of untreated or unstable medical condition such as poorly controlled diabetes mellitus, high blood pressure, renal disease, mental illness, HIV infection and other chronic infections (assessed through history taking from patients and documentation from case notes).

### Sample size determination

Cochran formula for sample size determination: *n* = *Z*^2^ *p q*/*d*^2^

where *n* is the sample size for populations greater than 10,000 people; *Z* = confidence interval is 1.96; *p* = prevalence (of fatigue in BCS = 24%) [11]; *q* = 1−*p* = 1− = 0.76 ; *D* = desired precision value = 0.05.

*n* is therefore (1.96)^2^ × 0.24 × 0.76/(0.05)^2^= 280

For populations less than 10,000, this formula is used: nf = n1 + n/N

where *n* (sample size for the population greater than 10,000)

*N* = study population = 98 (approximate number of women with BCS seen in 10 months)

*n f* = 280 ÷ (1 + 280/98) = 280 ÷ (1 + 2.86) = 73

A sample size of 80 participants was selected.

A total of 80 consecutive BCS who met the inclusion criteria were recruited for the study. Patients were recruited between 2 December 2019 and 2 October 2020. Data was collected using an interviewer-administered questionnaire after receiving their consent. Sociodemographic, disease and treatment characteristics were obtained from the participants as well as from their medical records. Fatigue was defined as tiredness, exhaustion or lack of energy [9, 18]. The third item on fatigue symptom inventory (FSI) was used to quantify average fatigue in the participants. The FSI is a tool that has been validated to assess fatigue in cancer patients as well as survivors [18]. It was previously used to compare the severity of fatigue in BCS and apparently healthy individuals in south-western Nigeria [11]. According to the FSI, participants were asked to rate their level of fatigue (tiredness, exhaustion or lack of energy) on average in the last week. Fatigue severity was assessed with 11-point items using the Likert scale (0 = not at all fatigued, 10 = as fatigued as I could be). The frequency of fatigue was taken as a score of 3 or greater on the scale. This is the recommended cut-off for discriminating cases of clinically meaningful fatigue [18, 19].

Descriptive statistics were presented, and appropriate tables were used. Mean and SD were used for variables that were normally distributed like age. Student *t*-test was used to compare means while chi-squared test was used for a comparison of proportions. Multiple linear regression analysis was used to adjust for confounders in statistically significant variables on univariate analysis. The level of significance was at 5%.

## Results

The patient’s sociodemographic variables and disease characteristics are shown in Table 1. The mean age of the recruited BCS was 51.36 ± 8.866 years The age of most of the patients was between 50 and 59 years (*n* = 44, 55%). The majority of the patients (*n* = 60, 75%) were married. As regards education status, (*n* = 49, 61.3%) had tertiary education, (*n* = 21, 26.3%) had secondary education while (*n* = 8, 10.0%) had primary education. Forty-six (57.5%) patients were employed at the time of recruitment. As regards the stage of disease as at the time of first visit, (*n* = 39, 48.8%) had stage 3 disease while (*n* = 41, 51.2%) had stage 2 disease. Thirty-two (40%) patients had comorbidities like hypertension and diabetes.

All the patients received chemotherapy, with the majority (*n* = 67, 83.8%) receiving anthracycline-based chemotherapy and (*n* = 62, 63.3%) receiving at least six courses. The period of last chemotherapy was within 1 year in (*n* = 34, 42.5%) patients. The prevalence of fatigue in this study was 22.5% (Table 1).

Table 2 shows sociodemographic and treatment-related correlates of fatigue in BCS. The mean age of BCS that experienced fatigue was significantly lower than those who did not (*p* = 0.022). Level of education was not associated with fatigue in these studies (*p* = 0.354). Patients that were employed however reported significantly higher fatigue (*p* = 0.012). Late stage of presentation (*p* = 0.014), previously receiving anthracycline chemotherapy (*p* = 0.026) and last year of chemotherapy within 1 year (*p* = 0.001) were associated with the occurrence of fatigue in BCS. The number of cycles received was however not significantly related with fatigue (*p* = 0.392) (Table 2).

Multivariate analysis of the correlates of fatigue in BCS was shown in Table 3. The results showed that stage at diagnosis (Odds ratio (OR) 5.115, 95% CI 1.029–25.438, *p* = 0.046), employment status (OR 52.224, 95% CI 3.611–755.899, *p* = 0.004) and year of last cycle of chemotherapy (OR 6.375, 95% CI 1.108–36.680, *p* = 0.038) were independent predictors of fatigue in BCS.

## Discussion

The prevalence of fatigue was 22.5% in this study. This is in keeping with the finding of a similar study that reported a prevalence of 24.3% [7]. However, a meta-analysis of 27 studies reported that the prevalence rates of fatigue in BCS ranged from 7% to 52% with a pool prevalence of 26.7% [10]. This shows that fatigue affects a large number of BCS and suggests the need to pay attention to this plight of BCS.

The study found that younger age was associated with fatigue in BCS. Patients who were also employed at the time of recruitment also reported more fatigue than the unemployed which agrees with the findings of similar studies [9, 10]. Survivors with CRF have a higher odds of being unemployed compared to non-cancer cohort and fatigue has been identified as a major barrier to returning to work following cancer treatment [20]. Unemployment or frequent absenteeism from work can result in wage loss and increase the risk of financial toxicity [21]. The fact that younger age was associated with fatigue is worrisome as young age is usually the productive age [22]. Survivors that were employed are more likely to be involved in physical activities which may worsen their baseline CRF [20]. Though healthy individuals may also experience fatigue while engaging in physical activities, CRF is more severe, persistent and disproportionate to recent activity [3, 4, 7]. This may contribute to the OR reported in the employed group reported in this study. The wide OR should however be interpreted with caution due to a small sample size. It is therefore desirable to further explore the relationship between CRF and the employment status of BCS using a larger sample size through a multi-institutional longitudinal study. This has the potential to shed more light to the economic impact of cancer diagnosis in Nigeria.

This study found that fatigue was not significantly related to educational status in BCS among the study population. This differs from the findings of some community-based studies which reported that apparently healthy people with higher level of education tend to have lesser fatigue [23, 24]. Similar study conducted on BCS reported divergent results [9, 13, 25]. The difference in the findings was linked to variations in sampling strategy, sample size, and study setting [10].

This study did not demonstrate the association between the presence of comorbidity and fatigue in BCS. This is at variance with the findings of some previous studies that showed worsening fatigue with hypertension, diabetes, arthritis and chronic pulmonary disease [14, 26]. A community-based cross-sectional study involving 1,158 apparently healthy individuals demonstrated that the presence of one or more chronic health problems was associated with fatigue [23]. The difference between this study and ours may be due to the fact that the survivors that was recruited in our study had no known history of untreated or unstable medical conditions such as poorly controlled diabetes mellitus, high blood pressure and renal disease. Though the patients were recruited at the radiation oncology clinic, their medical records contain information from other clinics they had visited within the hospital.

We also found that patients with stage 3 disease reported more fatigue than those with stage 2. This is in keeping with the findings of Abraham *et al* [13] that showed that early presentation is associated with less fatigue in BCS. Advanced disease is associated with more distressing symptoms and more adverse sequelae after treatment [27]. This further shows the importance of early presentation and prompt treatment in reducing the overall cancer burden not only at diagnosis and treatment stage but also during survivorship period [10].

The result from this study showed that chemotherapy was associated with fatigue. This agrees with the findings of a study that assessed the risk factors for chronic fatigue in BCS [13]. It was observed that patients who received anthracycline-based chemotherapy reported more fatigue than those who received non-anthracycline. Fatigue is a common side effect of anthracyclines [28]. The pathway for anthracycline-induced fatigue is not clear. Some authors demonstrated that anthracyclines produce reactive oxygen species that result in oxidative stress not only in cardiac muscle but also in skeletal muscle [29]. This drug-induced oxidative stress is a potential mechanism underlying the documented fatigue experienced by cancer patients [30]. Anthracyclines have also been implicated in cytokine-mediated neuroinflammation and brain damage in BCS which could be another potential mechanism for fatigue [28]. Though anthracyclines are classified as being unable to actively cross the blood-brain barrier, they are not completely excluded from the brain [31]. Even in small amounts, such agents can have clinically significant negative effects [31]. Further study would be necessary to further explore the mechanism of fatigue in patients taking anthracyclines.

We also observed that patients who completed their chemotherapy not more than a year reported more fatigue than those who completed it more than a year before the time of this study. This finding is in keeping with that of previous studies which demonstrated that the shorter the time since chemotherapy completion, the more fatigue experienced [9, 16, 32]. The first year after chemotherapy forms part of the period termed the re-entry phase in which patients confront psychosocial issues like fatigue and accompanying information needs [13, 33]. This further suggests the inclusion of fatigue in the follow up care of cancer survivors, especially in the first year after chemotherapy.

## Conclusion

About a quarter of BCS reported fatigue. The advanced stage of disease, employment status and recency of the last cycle of chemotherapy are correlates of fatigue. There should be a concerted effort in reducing the impact of fatigue in BCS. More attention should be paid to patients with features associated with fatigue in this study. Future studies will be necessary to investigate culturally accepted interventions that can reduce fatigue in BCS.

## Limitations

Fatigue was assessed in the survivors, months after treatment. The baseline pretreatment fatigue can have some influence on the post treatment findings. A prospective study that monitors fatigue before treatment, during treatment and at follow-up, could have addressed this limitation. Patients were also asked about fatigue they experienced in the last 7 days, which could predispose them to recall bias and under reporting of fatigue. The small sample size is another limitation of this study. There are potential confounders of fatigue such as pain, insomnia, patients’ physical activity and depression which were not considered in the study. Nonetheless, this study has provided baseline information that would be useful for a future longitudinal prospective study on CRF in BCS.

## Conflicts of interest

None.

## Funding

None.

## Ethics approval and consent to participate

Approval for this study was sought from the joint ethical review committee of the University of Ibadan/UCH, Ibadan.

## Consent for publication

Consent was taken from the patients with aid of informed consent form.

## Availability of data and material

The datasets used and/or analysed during the current study are available from the corresponding author on reasonable request.

## Author contributions

SAF: Conceptualised the topic and designed the study methodology. He contributed to the data acquisition, data analysis, interpretation of data and wrote the final draft of this work.

AAS: Contributed to the conception, design, data acquisition, data analysis, interpretation of data and the draft of this work.

AIN: Contributed to the conception, design, data acquisition, data analysis, interpretation of data and the draft of this work.

AAO: Contributed to the conception, design, data acquisition, data analysis, interpretation of data and the draft of this work.

MAJ: Contributed to the conception, design, data acquisition, data analysis, interpretation of data and the draft of this work.

AOF: Contributed to the conception, design, data acquisition, data analysis and the draft of this work.

They have approved the submitted version and the final draft of the manuscript and take responsibility for this paper.

## List of abbreviations

BCS, Breast cancer survivors; CRF, Cancer related fatigue; FSI, Fatigue symptom inventory; UCH, University College Hospital Ibadan.

## Figures and Tables

**Table 1.** Patient’s sociodemographic variables, disease characteristics, treatment information and prevalence of fatigue.

Variable	Category	Frequency	Percentage
Age			
	<30 years	3	3.8
	30–39 years	3	3.8
	40–49 years	17	21.3
	50–59 years	44	55.0
	60–69 years	12	15.0
	≥70 years	1	1.3
Marital status			
	Married	60	75.0
	Single	4	5.0
	Divorced	2	2.5
	Widowed	14	17.5
Education			
	Primary	8	10.0
	Secondary	21	26.3
	Tertiary	49	61.3
	No formal Education	2	2.5
Employment status			
	Presently employed	46	57.5
	Unemployed	34	39.8
Stage at presentation			
	2	39	48.8
	3	41	51.2
Presence of comorbidity			
	Yes	32	40.0
	No	48	60.0
Chemotherapy regime			
	Anthracycline-based	67	83.8
	Non-anthracycline-based	13	16.3
Number of chemotherapy cycles			
	≤6 cycles	67	83.8
	>6 cycles	13	16.2
Year of last cycle of chemotherapy			
	≤1 year	34	42.5
	>1 year	46	57.5
Prevalence of fatigue		18	22.5

**Table 2.** Univariate analysis of the sociodemographic and treatment-related correlates of fatigue in breast cancer survivors.

Variable	Categories	Fatigued	Not fatigued	*p* value
Age	(Mean ± SD)	48.67 ± 4.379	52.34 ± 9.183	0.022
Marital status				
	Married	12	48	0.354
	Others	6	14	
Education				
	Primary or no formal education	2	5	0.687
	At least secondary education	16	57	
Employment status				0.012
	Employed	15	31	
	Unemployed	3	31	
Stage at presentation				0.014
	2	4	35	
	3	15	26	
Presence of comorbidity				0.325
	Yes	9	23	
	No	9	39	
Chemotherapy regime previously received				0.026
	Anthracycline	12	55	
	Non anthracycline	6	7	
Number of chemotherapy cycle				0.392
	≤6 cycles	9	38	
	>6 cycles	9	24	
Year of the last chemotherapy				0.001
	≤I year	14	20	
	>1 year	4	42	

**Table 3.** Multivariate analysis of the correlates of fatigue in breast cancer survivors.

Variable	OR	*p*-value	95% Confidence interval	
			Lower	Upper
Age (younger age)	0.921	0.154	0.823	1.031
Stage (Stage 3)	5.115	0.046	1.029	25.438
Number of cycles (≤6 cycles)	1.691	0.489	0.382	7.475
Employment status (Employed)	52.224	0.004	3.611	755.899
Chemotherapy regime (Anthracycline)	4.021	0.280	0.322	50.215
Year of chemotherapy (≤1 year)	6.375	0.038	1.108	36.680

## References

[1] Sung H, Ferlay J, Siegel RL (2021). Global cancer statistics 2020: GLOBOCAN estimates of incidence and mortality worldwide for 36 cancers in 185 countries. CA Cancer J Clin.

[2] Hassan H, Bayoumi M, Atwa A (2016). Emotional distress associated with gynecologic and breast cancer in Beni-Suef City. Int J Sci Res IJSR.

[3] Francies FZ, Hull R, Khanyile R (2020). Breast cancer in low-middle income countries: abnormality in splicing and lack of targeted treatment options. Am J Cancer Res.

[4] Folasire A, Folorunso S, Orekoya A (2020). Pattern and outcome of admission of cancer patients at radiation oncology ward University College Hospital Ibadan Nigeria. J Radiat Med Trop.

[5] Ngwa W, Addai BW, Adewole I (2022). Cancer in sub-Saharan Africa: a Lancet Oncology Commission. Lancet Oncol.

[6] Miller KD, Siegel RL, Lin CC (2016). Cancer treatment and survivorship statistics, 2016. CA Cancer J Clin.

[7] Bower JE (2008). Behavioral symptoms in patients with breast cancer and survivors. J Clin Oncol.

[8] Neefjes ECW, Vorst MJDL, Blauwhoff-Buskermolen S (2013). Aiming for a better understanding and management of cancer-related fatigue. Oncologist.

[9] Prue G, Rankin J, Allen J (2006). Cancer-related fatigue: a critical appraisal. Eur J Cancer.

[10] Abrahams HJG, Gielissen MFM, Schmits IC (2016). Risk factors, prevalence, and course of severe fatigue after breast cancer treatment: a meta-analysis involving 12 327 breast cancer survivors. Ann Oncol.

[11] Folorunso SA, Ntekim A, Abdus-Salam AA (2022). Fatigue as a complicating factor in the recovery of breast cancer survivors treated at an oncology clinic in South West Nigeria: a case-control study. Ecancermedicalscience.

[12] Wang XS (2008). Pathophysiology of cancer-related fatigue. Clin J Oncol Nurs.

[13] Abrahams HJG, Gielissen MFM, Verhagen CAHHVM (2018). The relationship of fatigue in breast cancer survivors with quality of life and factors to address in psychological interventions: a systematic review. Clin Psychol Rev.

[14] Schmidt ME, Chang-Claude J, Seibold P (2015). Determinants of long-term fatigue in breast cancer survivors: results of a prospective patient cohort study: determinants of long-term fatigue in breast cancer survivors. Psychooncology.

[15] Cavalli Kluthcovsky ACG, Urbanetz AA, Carvalho DS (2012). Fatigue after treatment in breast cancer survivors: prevalence, determinants and impact on health-related quality of life. Support Care Cancer.

[16] Servaes P (2002). Determinants of chronic fatigue in disease-free breast cancer patients: a cross-sectional study. Ann Oncol.

[17] Njoku MGC, Jason LA, Torres-Harding SR (2007). The prevalence of chronic fatigue syndrome in Nigeria. J Health Psychol.

[18] Donovan KA, Jacobsen PB (2011). The fatigue symptom inventory: a systematic review of its psychometric properties. Support Care Cancer.

[19] Hann DM, Jacobsen PB, Azzarello LM (1998). Measurement of fatigue in cancer patients: development and validation of the fatigue symptom inventory. Qual Life Res Int J Qual Life Asp Treat Care Rehabil.

[20] Jones JM, Howell D, Longo C (2023). The association of cancer-related fatigue on the social, vocational and healthcare-related dimensions of cancer survivorship. J Cancer Surviv Res Pract.

[21] Wolvers MDJ, Leensen MCJ, Groeneveld IF (2019). Longitudinal associations between fatigue and perceived work ability in cancer survivors. J Occup Rehabil.

[22] Mbonigaba J, Akinola WG (2021). Productivity and income effect of breast cancer among women in Southwestern Nigeria. Economies.

[23] Jing MJ, Wang JJ, Lin WQ (2015). A community-based cross-sectional study of fatigue in middle-aged and elderly women. J Psychosom Res.

[24] Junghaenel DU, Christodoulou C, Lai JS (2011). Demographic correlates of fatigue in the US general population: results from the patient-reported outcomes measurement information system (PROMIS) initiative. J Psychosom Res.

[25] Wagner LI, Cella D (2004). Fatigue and cancer: causes, prevalence and treatment approaches. Br J Cancer.

[26] Groenvold M, Petersen MA, Idler E (2007). Psychological distress and fatigue predicted recurrence and survival in primary breast cancer patients. Breast Cancer Res Treat.

[27] Delgado-Guay MO, Ferrer J, Ochoa J (2018). Characteristics and outcomes of advanced cancer patients who received palliative care at a public hospital compared with those at a comprehensive cancer center. J Palliat Med.

[28] Kesler SR, Blayney DW (2016). Neurotoxic effects of anthracycline- vs nonanthracycline-based chemotherapy on cognition in breast cancer survivors. JAMA Oncol.

[29] van Norren K, Helvoort A, Argilés JM (2009). Direct effects of doxorubicin on skeletal muscle contribute to fatigue. Br J Cancer.

[30] Gilliam LAA, Clair DKS (2011). Chemotherapy-induced weakness and fatigue in skeletal muscle: the role of oxidative stress. Antioxid Redox Signal.

[31] Rousselle C, Smirnova M, Clair P (2001). Enhanced delivery of doxorubicin into the brain via a peptide-vector-mediated strategy: saturation kinetics and specificity. J Pharmacol Exp Ther.

[32] Fabi A, Falcicchio C, Giannarelli D (2017). The course of cancer related fatigue up to ten years in early breast cancer patients: what impact in clinical practice?. Breast.

[33] Stanton AL (2006). Psychosocial concerns and interventions for cancer survivors. J Clin Oncol.

